# Nanoindentation of embedded particles

**DOI:** 10.1557/s43578-023-00920-2

**Published:** 2023-02-10

**Authors:** Alejandra Slagter, Joris Everaerts, Andreas Mortensen

**Affiliations:** 1 Mechanical Metallurgy Laboratory, École Polytechnique Fédérale de Lausanne, MXD 140 (Bâtiment MXD), Station 12, 1015 Lausanne, Switzerland; 2 Present Address: Department of Materials Engineering, KU Leuven, Kasteelpark Arenberg 44 Box 2450, 3001 Leuven, Belgium

**Keywords:** Nano-indentation, Elastic properties, Hardness, Composite

## Abstract

We address the effect of elastic inhomogeneity on elastic modulus and hardness determinations made by depth-sensing indentations performed on individual particles embedded within a matrix of different elastic modulus. Finite element simulations and nanoindentation experiments are used to quantify the consequences of particle/matrix elastic inhomogeneity and we propose an adaptation of the Oliver–Pharr method that gives access to particle properties knowing those of the matrix. The method is suitable for any combination of matrix and particle elastic modulus and for any type of indenter, provided that the area of the tested particles along the surface of the sample is measured and that a large number of particles are probed. Further conditions for the implementation of the method are that testing conditions be such (i) that permanent deformation of the matrix is avoided, and (ii) that permanent deformation in each probed particle under the indenter is not affected by the matrix.

**Graphical abstract:**

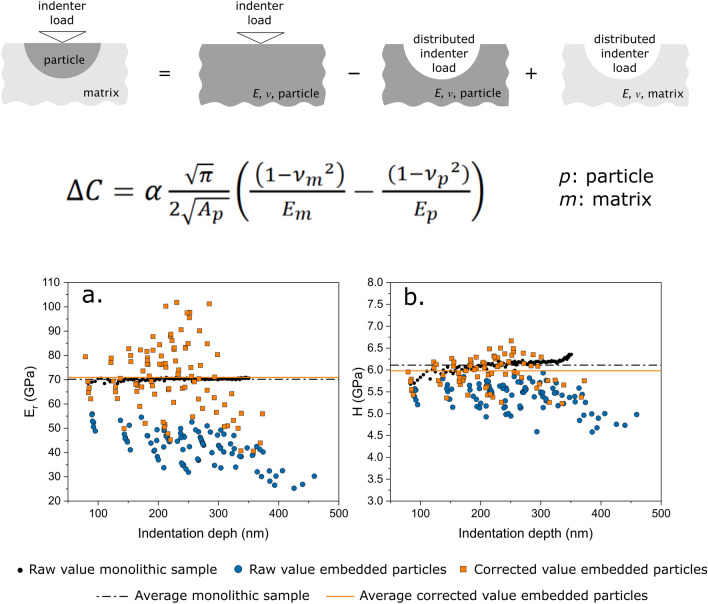

## Introduction

Instrumented indentation, or nanoindentation experiments, provide a rich source of information on the local mechanical behavior of materials. Derived properties, such as the hardness (*H*) and elastic modulus (*E*), can be calculated with good accuracy using well-established schemes such as that of Oliver and Pharr [1], which builds on a solid understanding of the factors affecting measured loads and displacements when indenting a semi-infinite continuum with a hard probe of defined geometry.

Data interpretation schemes designed for instrumented nanoindentation rely, however, on several assumptions; among those are: (i) the probed material is assimilated to a homogeneous, semi-infinite solid, and (ii) knowledge of the indenter elastic properties is required. Yet, for many of the situations where nanoindentation is interesting, such as probing the properties of small volumes of material or thin films, those assumptions are not met and, as a consequence, specific testing strategies and/or correction techniques need to be implemented in order to obtain meaningful data.

A vast body of literature can thus be found that is concerned with the extraction of elastic modulus and hardness values from films deposited on various substrates. Interest in using nanoindentation to extract information on the mechanical properties of films on substrates is almost as old as the instrumented indentation technique itself. The first model to correct for substrate effects when measuring the elastic modulus was based on empirical observations and was proposed by Doerner and Nix [2] in 1986. The work of Gao et al. [3] followed a few years later with a model based on moduli-perturbation analysis for multilayered materials and, over the years, numerous authors have addressed the subject, proposing simpler models, studying the range of validity of existing models, or optimizing them for certain testing conditions or types of material [4–8].

Comparatively, the extraction of the elastic properties of particles embedded in a matrix of different stiffness has been far less explored; yet, the question is relevant to many situations, such as our understanding of multiphase material micromechanics or probing phases of small volume. Experimental approaches have been proposed by Jakes et al. [9], and Buchheit and Vogler [10], who suggested a correction method based on the determination of a ‘structural’ or ‘system’ compliance. The methods generally assume a constant ratio of hardness to modulus squared for the tested material and determine the local extra compliance induced by the surrounding matrix at each indentation location by using either a multiple unloading indentation strategy or a continuous stiffness measurement (CSM) technique.

The problem has also been addressed through numerical simulation. Based on finite element models of indentations performed with a conical indenter on a semi-spherical particle, Yan et al. [11, 12] have defined a particle-dominated depth over which the results of elastic modulus determinations are affected to less than 10% by matrix effects. Their results indicate that the particle-dominated depth depends largely on the ratio of the elastic modulus of the particle to that of the matrix and suggest that the indentation depth should be kept to less than 5% of the particle radius. Other finite element simulations and experimental measurements were performed by Leggoe [13], with focus on ceramic reinforcements in metal matrix composites (MMC). It was found that, for indentations performed using a spherical indenter (and for the particle geometries and range of properties analyzed), the total displacement experienced by the indenter is (i) what the particle material alone would yield were it to be sufficiently large and not surrounded by the matrix, plus (ii) a term accounting for the presence of the matrix. The latter, namely the additional penetration ($\Delta u)$ of the indenter caused by the presence of the matrix at load *P,* is inversely proportional to the particle diameter (${D}_{\mathrm{p}})$ and is related to the elastic modulus of matrix (sub-index *m*) and particle (sub-index *p*) by:

1 $$\frac{\Delta u}{P}=\alpha \frac{1}{{D}_{p}}\left(\frac{ \left(1-{{\nu }_{m}}^{2}\right)}{{E}_{m}}-\frac{ (1-{{\nu }_{p}}^{2})}{{E}_{p}}\right),$$

where $\alpha$ is a proportionality constant, estimated from finite element simulations to be 0.925 for spherical particles (embedded to the equator) and 0.802 for cylindrical particles of depth equal to their diameter.

Finally, the problem was addressed by Lamagnere et al. [14] who studied, by means of nanoindentation, the hardness and elastic modulus of non-metallic inclusions embedded in a steel matrix. Their approach to the problem was to demonstrate, by finite element simulations, that the effect of the matrix on the indentation experiments did not cause deviations that exceeded 5% of the elastic modulus value, particularly for the range of dimensions of their non-metallic inclusions (assuming those have a spherical geometry) and for the expected elastic properties of particle and matrix. An interesting aspect of their work is that they considered in their simulations particles embedded to different depths (¼, ½, and ¾ of a sphere) and showed that the effect of the matrix on the nanoindentation results would depend not only on the real particle radius but also on its relative depth into the matrix.

We show in the following that an equation close to Eq. (1) can be used to calculate an average extra compliance produced by the presence of a matrix around the particle, which can be used to correct both hardness and elastic modulus values gathered after indenting a sufficient number of particles. This extra compliance can be estimated regardless of the type of indenter used and regardless of the difference in elastic properties between matrix and particle. We thus extend Leggoe’s results to include spherical and cylindrical particles embedded to different depths, we analyze the conditions under which the extra compliance calculation remains valid and we show that a key condition for the approach to be valid is that a sufficient number of particles, on the order of 30 or more, be tested.

## Background

### Problem statement

In instrumented indentation experiments, the most generally derived quantities are the hardness, *H*, and the reduced elastic modulus, ${E}_{\mathrm{r}}$, of the probed material. These are calculated as:

2 $$H=\frac{P}{{A}_{\mathrm{c}}},$$

3 $${E}_{\mathrm{r}}=\frac{1}{\beta }\frac{S\sqrt{\pi }}{2\sqrt{{A}_{\mathrm{c}}}},$$

where the indentation load (*P*) is directly measured during the test, while the unloading stiffness (*S*) and the corresponding projected contact area (${A}_{\mathrm{c}}$) are derived from the load–displacement curve. Parameter $\beta$, which can be taken equal to 1.05 [15], is a constant introduced to correct for deviations from axial symmetry in the indenter geometry. The reduced modulus ${E}_{\mathrm{r}}$ is a combination of the elastic properties of the indenter (sub-index *i*) and indented material (sub-index *s*) defined by:

4 $$\frac{1}{{E}_{\mathrm{r}}}=\frac{1-{\nu }_{i}^{2}}{{E}_{i}}+\frac{1-{\nu }_{s}^{2}}{{E}_{s}}.$$

Oliver and Pharr pointed out that, while the load is being removed, the contact area gradually changes. As a result, the unloading curve is not linear but is better described by a power law function of the form:

5 $$P=A{(h-{h}_{f})}^{m},$$

where *A* and *m* are fitting parameters, ${h}_{f}$ is the final indentation depth, and *h* is the indenter tip displacement at load *P*. The unloading stiffness is defined as the derivative of Eq. (5) computed at maximum load:

6 $$S={\left.\frac{\mathrm{d}P}{\mathrm{d}h}\right|}_{P={P}_{\mathrm{max}}}.$$

Oliver and Pharr determined the contact depth (${h}_{\mathrm{c}}$) from the difference between the maximum depth measured during the indentation test (${h}_{\mathrm{max}}$) and the elastic sink-in of the sample surface around the indenter. In the absence of pile-up formation, the latter is expressed using Sneddon’s solution for the deformation of a half space under a conical indenter. This leads to the following expression for ${h}_{\mathrm{c}}$ [1]:

7 $${h}_{\mathrm{c}}={h}_{\mathrm{max}}-\epsilon \frac{P}{S},$$

where $\epsilon$ is a constant of value $\epsilon$ = 0.75 [1, 15].

Accurate determination of the contact depth allows the contact area to be computed after calibration of the tip with a reference material of known elastic modulus. The contact area, ${A}_{\mathrm{c}}$, is usually described as a function of the contact depth $({h}_{\mathrm{c}}$) by a function of the form:

8 $${A}_{\mathrm{c}}={C}_{0}{h}_{\mathrm{c}}^{2}+{C}_{1}{h}_{\mathrm{c}}+{C}_{2}{h}_{\mathrm{c}}^{1/2}+{C}_{3}{h}_{\mathrm{c}}^{1/4}+{C}_{4}{h}_{\mathrm{c}}^{1/8},$$

where all ${C}_{i}$ are fitting parameters, with ${C}_{0}$ usually taken as 24.5 for a Berkovich indenter.

When probing the properties of a particle embedded in a semi-infinite material with different elastic properties, there is an extra (positive or negative) displacement induced by the matrix. This has an effect on both *S* and ${h}_{\mathrm{c}}$ and, if not accounted for, it leads to an inaccurate determination of both the elastic modulus and nanoindentation hardness.

### Estimating the matrix-induced change in compliance

In Ref. [13] Leggoe proposes viewing the influence exerted by the matrix on data collected by testing embedded particles as being a ‘secondary indentation’ in the sense that, when probed by nanoindentation, the hard and stiff ceramic reinforcements of metal matrix composites that were probed in his work could be seen to act as indenters themselves, pressed against the more compliant matrix material. In the following, we build on this idea to derive a (more) general expression for the influence exerted by the surrounding matrix on nanoindentation data collected by indenting, at sufficiently low loads, embedded particles in a two-phase particulate material.

The derivation considers only elastic contributions from material situated below the particle material (where deformations are expected to be small) and builds on the principle of superposition of elastic deformations. The resulting equation is thus valid only if (i) the zone of permanent deformation around the indenter is confined to the indented particle in such a way that its size and characteristics are not affected by the presence of the matrix, and if (ii) the indentation load is kept sufficiently low such that the matrix does not deform permanently. Condition (i) is generally satisfied if the indentation is kept from the particle–matrix interface at a distance greater than ten times the indentation depth [16]. Condition (ii) requires that the indentation load divided by the in-plane particle surface area remain well below the matrix hardness (or three times the matrix uniaxial yield stress [8]). Under these assumptions, since the extra deformation coming from the matrix is purely elastic, the problem can be analyzed in terms of compliances (or displacements at fixed load).

**Figure 1:**
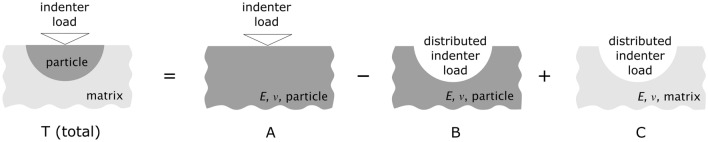
Schematic representation of the idealized sub-systems into which the total compliance (*C*_T_) of an indented particle embedded within an elastically different matrix can be divided (see text for details).

We thus decompose the indenter-particle–matrix system into the addition of smaller ideal sub-systems (Fig. 1). In this construction, the short-range deformations (elastic and plastic) immediately around the sharp indenter are considered to be identical (i) in a semi-infinite homogeneous sample entirely made of particle material (A in Fig. 1) and (ii) in the embedded particle (T in Fig. 1). Beyond the particle/matrix interface, all strain is assumed to be well within the range of linear elastic deformation in either A or T of Fig. 1. Given that the material in these two systems differs, strain fields beyond the particle/matrix interface are not the same in A or T of Fig. 1.

Having assumed that the difference between the indented embedded particle and a semi-infinite material with particle properties is to be found in the material (and ensuing deformation) beyond the particle, then the total displacement of the indenter tip in T can be expressed as the total displacement in A minus the displacement in B (this difference represents the total tip displacement in T due only to strain within the particle), plus the displacement in C. The total system compliance, (the inverse of its stiffness *S*) can then be expressed as the following linear combination of the compliance of each of the three sub-systems:

9 $${C}_{\mathrm{T}}={C}_{\mathrm{A}}-{C}_{\mathrm{B}}+{C}_{\mathrm{C}}.$$

To (approximately) compute compliances in B and C, we assume that the probed particles can themselves be considered to act as an indenter (the nature of which is unimportant since terms related to its elastic constants cancel out in ${C}_{\mathrm{C}}-{C}_{\mathrm{B}},$ see Eqs. 3 and 4).

The terms in Eq. (9) can then be described as follows:

${C}_{\mathrm{A}}$ is the compliance of a uniform semi-infinite solid having properties of the particle, when it is indented under the same indenter and conditions as the embedded particle;

${C}_{\mathrm{B}}$ is the compliance that would be displayed by a uniform semi-infinite solid having properties of the particle when it is probed with an indenter of shape and dimensions equal to those of the particle;

${C}_{\mathrm{C}}$ is the compliance of a uniform semi-infinite solid with properties of the matrix when it is being probed with an indenter of shape and dimensions equal to those of the particle.

In other terms, the elastic contribution of the matrix results in an “extra” (positive or negative) compliance ∆*C* which can be defined as the difference between the compliance observed experimentally in the composite system, ${C}_{\mathrm{T}}$, and the compliance, ${C}_{\mathrm{A}}$, that would be observed in a semi-infinite (homogeneous) material having the unknown particle elastic properties:

10 $$\Delta C={C}_{\mathrm{T}}-{C}_{\mathrm{A}}.$$

Note that ${C}_{\mathrm{T}}$ can be measured experimentally while ${C}_{\mathrm{A}}$ is the compliance-corrected value that would lead to an accurate determination of the elastic modulus of the particle material alone following established schemes such as the Oliver–Pharr method. Under present assumptions, from Eq. (9):

11 $$\Delta C=-{C}_{\mathrm{B}}+{C}_{\mathrm{C}}.$$

The assumption made here, namely that the particle acts as an indenter in the matrix, is essentially equivalent to considering that the particles are: (i) infinitely rigid (the imposed particle displacement field is uniform); (ii) have a frictionless interface with the matrix (only forces that are perpendicular to the particle–matrix interface and that push on the matrix are transferred); and (iii) that the particles are much smaller than the composite sample. If these approximations can be made then the compliance (*C*) of each of the two ideal sub-systems B and C in Fig. 1 can be written as:

12 $$C=\alpha \frac{\sqrt{\pi } (1-{\nu }^{2})}{2\sqrt{A }E},$$

where *E* and $\nu$ denote the Young’s modulus and Poisson’s coefficient of the indented material, and *A* denotes the projected contact area of the particle assimilated to an indenter. Note that Eq. (12) has naturally the same form as Eq. (3), but written in terms of the compliance (*C*) instead of the stiffness (*S*), since *S* = 1/*C*. We have also replaced the factor $1/\beta$ (of Eq. 3) by a slightly more general parameter $\alpha$ characteristic of the indenter ($\beta$ is a constant traditionally considered in the literature to account specifically for the lack of axial symmetry of a pyramidal indenter). In Eq. (12) $\alpha$ is then a proportionality constant, theoretically equal to unity for a uniform displacement imposed with a punch of axisymmetric geometry, but whose value will vary if, for example, the indented particles (acting as indenters themselves) do not present axial symmetry [4, 17], or if the interfacial pressure distribution deviates from that corresponding to an imposed uniform displacement [18].

An underlying assumption in the present derivation is that the remaining matrix material can be regarded as a homogeneous and continuous solid. In a material where the particles represent a minority phase, the matrix and surrounding particles can generally be assimilated to the matrix. If the particles represent a large fraction of the material, then the matrix will probably be better represented by the composite material. In what follows we consider the former case and thus take the matrix material past the particle/matrix interface to be the (unreinforced) matrix. For a highly loaded two-phase material the approach proposed here remains unchanged; however, iteration might have to be used to deduce particle properties since the matrix elastic modulus is calculated using particle properties. Alternatively, one might in such a case use results from measurements of the composite elastic properties, i.e., by indenting the particle/matrix composite with a probe much larger than the particles to define the matrix compliance to be used in computations.

Substituting Eq. (12) into Eq. (11), the following expression for the extra compliance is obtained:

13 $$\Delta C=\alpha \frac{\sqrt{\pi }}{2\sqrt{{A}_{p} }}\left(\frac{ \left(1-{{\nu }_{m}}^{2}\right)}{{E}_{m}}-\frac{ (1-{{\nu }_{p}}^{2})}{{E}_{p}}\right),$$

where ${A}_{p}$ is the projected contact area of the particle (acting as a secondary indenter), and subscripts *p* and *m* designate particle and matrix respectively.

For particles with a circular cross section, the term $\frac{\sqrt{\pi }}{2\sqrt{{A}_{p}}}$ is equivalent to $\frac{1}{{D}_{p}}$, with ${D}_{p}$ the diameter of the particle cross-section visible along the surface of the sample. Equation (13) then takes the same form as that proposed by Leggoe [13] (Eq. 1).

In summary, the above derivation shows that the extra compliance resulting from the difference in stiffness between the particle and its surroundings depends on the matrix and particle elastic constants, and on the dimensions of the probed particle through the square root of its projected contact area. Results of the above derivation are, within assumptions made, valid for indentations performed with any indenter and for any combination of elastic modulus, as long as particle indentation itself is not affected and the particle can be seen as a secondary indenter.

## Corrected computation of *E* and *H*

Corrected values for *E* and *H* can be computed after determination of the extra compliance, $\Delta C$, caused by the presence of the matrix. As expressed by Eq. (13), $\Delta C$ can be calculated knowing the particle dimensions and the elastic properties of both particle and matrix. The matrix reduced modulus, if not already known, can generally be probed directly by nanoindentation of the matrix material, provided that it can be obtained free of particles, or if the volume fraction of particles is sufficiently low such that the presence of the particles does not significantly affect matrix indentation. Determining the particle elastic modulus, however, is usually the aim of the indentation measurements, and therefore an accurate value of $\Delta C$ is generally not immediately available. In this situation, the average initial (raw) value of the particle reduced modulus obtained by probing embedded particles can serve to compute an initial value for $\Delta C$. This initial value can then be used to make a first estimation of the corrected elastic modulus, and this value can once more serve to calculate a second, more accurate, approximation of the elastic modulus. Following this procedure, iterations can be performed until the difference between corrected elastic modulus values before and after the last iteration falls below a chosen value.

Once $\Delta C$ is known (or estimated by iteration), then the unloading stiffness, ${S}^{\mathrm{corr}},$ can be simply corrected as:

14 $${S}^{\mathrm{corr}}={\left(\frac{1}{S}-\Delta C\right)}^{-1}.$$

The contact depth can then be corrected, again on the hypothesis that the short-range displacements around the indenter are not modified by the presence of the matrix. The measured displacement is then the sum of displacements caused by (i) permanent deformation within the indented material plus (ii) displacements emanating from the elastic sink-in of the indenter into the particle and (iii) elastic sink-in of the particle into the matrix. Under this assumption, and if the elastic sink-in of the matrix around the particle can still be described as if the particle was an indenter (that is, if Sneddon’s solution for the displacements around an axisymmetric indenter is also valid for the particle indenting the matrix), then the corrected contact depth ${h}_{\mathrm{c}}^{\mathrm{corr}}$ can be computed, following Eq. (7), as:

$${h}_{\mathrm{c}}^{\mathrm{corr}}={h}_{\mathrm{max}}^{\mathrm{corr}}-\epsilon P({C}^{\mathrm{corr}}),$$

where both terms in the right-hand side are corrected for the extra compliance, namely ${h}_{\mathrm{max}}^{\mathrm{corr}}={h}_{\mathrm{max}}-P\Delta C,$ and ${C}^{\mathrm{corr}}= C-\Delta C$. Therefore:

$${h}_{\mathrm{c}}^{\mathrm{corr}}={(h}_{\mathrm{max}}-P\Delta C)-\epsilon P(C-\Delta C),$$

$${h}_{\mathrm{c}}^{\mathrm{corr}}={h}_{\mathrm{max}}-\epsilon PC+\epsilon P\Delta C-P\Delta C,$$

15 $${h}_{\mathrm{c}}^{\mathrm{corr}}={h}_{\mathrm{c}}-P\Delta C(1-\epsilon ),$$

where ${h}_{\mathrm{c}}$ is the raw value for the contact depth (calculated from ${h}_{\mathrm{max}}$ according to Eq. 7). With this corrected value of the indenter/particle contact depth, a corrected value of the contact area (${A}_{\mathrm{c}}^{\mathrm{corr}}$) can in turn be computed using the contact area expression (Eq. 8) and parameters corresponding to the tip area calibration. Corrected values for the reduced modulus and the hardness of the probed material can then be calculated as:

16 $${E}_{\mathrm{r}}^{\mathrm{corr}}=\frac{1}{\beta }\frac{{S}^{\mathrm{corr}}\sqrt{\pi }}{2\sqrt{{A}_{\mathrm{c}}^{\mathrm{corr}}}},$$

17 $$H^{{{\text{corr}}}} = \frac{P}{{A_{{\text{c}}}^{{{\text{corr}}}} }}.$$

## Finite element calculations

The preceding derivation aims to account for the influence exerted by elastic inhomogeneity on indentation data generated when probing individual particles embedded within a two-phase material. Its premise is, following the derivation by Leggoe [13], that indented particles can be viewed as a second large indenter that probes its surrounding material viewed as an elastic continuum. Equation (13) makes no assumption regarding the shape of the particle; however, this is bound to exert an influence. Leggoe [13] assimilated the particle to a half-sphere, or to a cylinder as tall as its radius; however, even if the particles can be assimilated to spheres, there is a difference between the response of a hemisphere and that of a shallow spherical shell or a nearly whole embedded sphere, as shown by [14].

Finite element models were therefore constructed to simulate variously embedded isotropic linear elastic spherical particles, as well as cylinders of different aspect ratio and axis normal to the plane of polish, pushed into a large body of surrounding homogeneous isotropic linear elastic “matrix” material. In the simulations, a fixed displacement was imposed on the punch while the resultant reaction force in the direction of the imposed displacement was used to calculate the compliance of composite systems (${C}_{\mathrm{T}}$) and monolithic systems (${C}_{\mathrm{A}}$). The difference between ${C}_{\mathrm{T}}$ and ${C}_{\mathrm{A}}$ was computed for different particle elastic modulus values and represents a determination of Δ*C* for each simulated particle geometry, size, and combination of particle and matrix elastic properties. These values were subsequently made dimensionless to highlight the fact that obtained results are not limited to the chosen particle dimensions and elastic modulus in the simulations, but rather scale with those. We therefore made dimensionless the finite element results by the elastic properties of the particle and the diameter of the particle along the surface of the sample $({D}_{\mathrm{p}}$), such that:

18 $${\Delta C}^{\mathrm{adim}}=\Delta C {D}_{\mathrm{p}}\frac{{E}_{\mathrm{p}}}{(1-{{\upnu }_{\mathrm{p}}}^{2})}.$$

And therefore Eq. (13) can be expressed in dimensionless form by combining Eqs. (13) and (18) to give:

19 $${\Delta C}^{\mathrm{adim}}=\alpha \left(\frac{(1-{{\nu }_{\mathrm{m}}}^{2}){E}_{p}}{\left(1-{{\nu }_{\mathrm{p}}}^{2}\right){E}_{\mathrm{m}}}-1\right).$$

## Results

**Figure 2:**
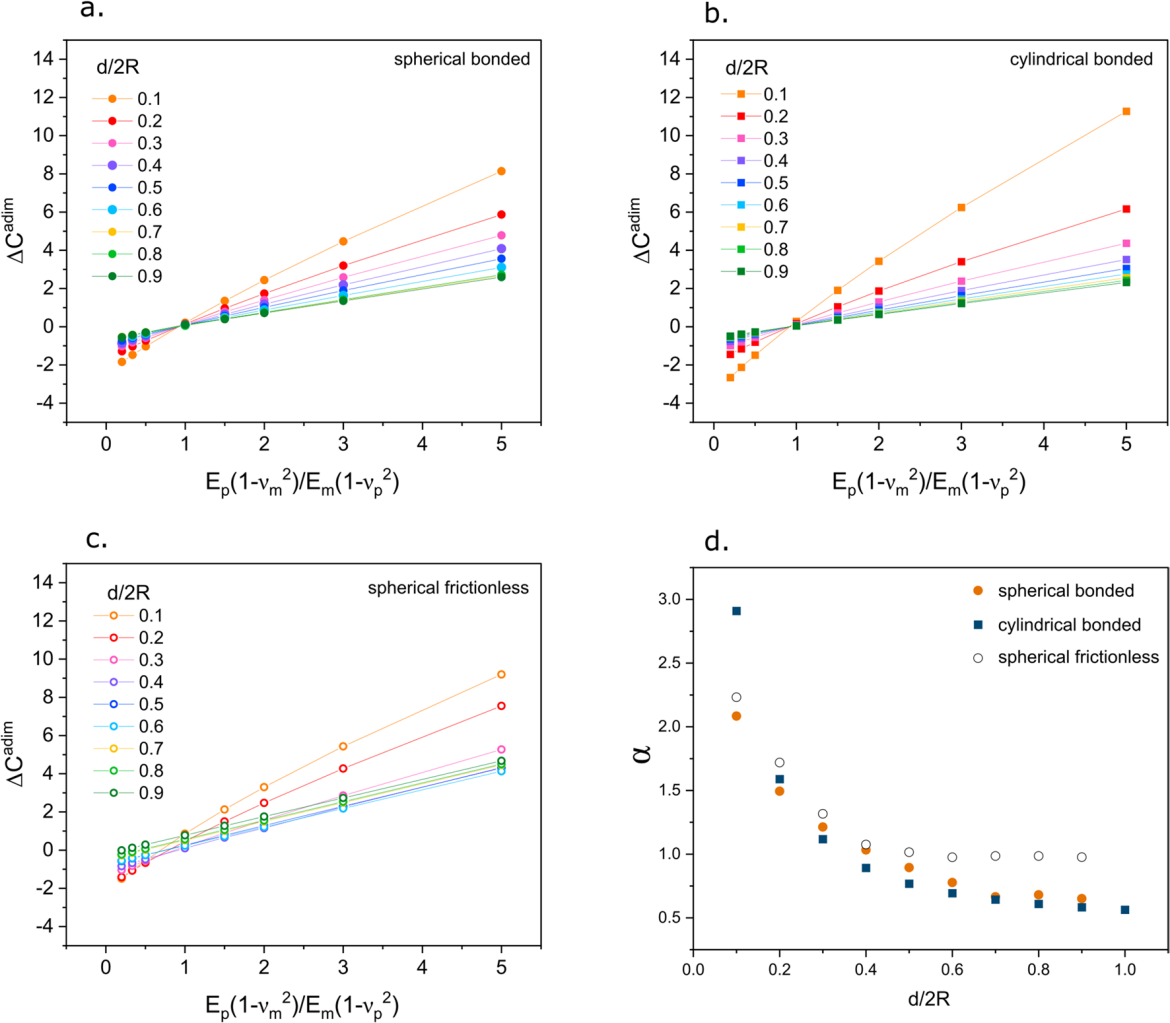
(a)–(c) dimensionless compliance ${(\Delta C}^{\mathrm{adim}}$) versus $\frac{(1-{{\nu }_{\mathrm{m}}}^{2}){E}_{\mathrm{p}}}{\left(1-{{\nu }_{\mathrm{p}}}^{2}\right){E}_{\mathrm{m}}}$, computed from finite element simulations of particles embedded to different relative depths in an isotropic elastic matrix having different elastic constants (depths, *d,* expressed as a fraction of the particle diameter, *2R*). (a) spherical particles with a perfectly bonded interface; (b) cylindrical particles with a perfectly bonded interface; (c) spherical particles with a frictionless interface; (d) value of the slope obtained by minimum square fit of lines in (a) to (c).

Figure 2 presents results of the finite element simulations, in a plot of the dimensionless compliance, ${\Delta C}^{\mathrm{adim}}$ versus $\frac{(1-{{\nu }_{\mathrm{m}}}^{2}){E}_{\mathrm{p}}}{\left(1-{{\nu }_{\mathrm{p}}}^{2}\right){E}_{\mathrm{m}}}$ (see Eq. 19) for (a) spheres, (b) cylinders, both with a fully coherent interface, and (c) spheres assuming a frictionless interface.

Deduced values of α, calculated by a least-squares fit of points in Fig. 2(a–c) are plotted in Fig. 2(d) versus the ratio of the particle depth (*d*) to diameter (*2R*). As seen, α varies with the particle relative depth but remains essentially the same regardless of whether the particles are spherical or cylindrical, or are spheres loosely bonded to the matrix except, in this last case, for particles embedded to less than the equator, for which the value of alpha remains approximately equal to unity.

## Discussion and experimental implementation

Finite element simulations show that Eq. (13) (or in dimensionless form Eq. 19) adequately captures, for simulated geometries, the extra compliance caused by elastic modulus inhomogeneity between the surrounding matrix and the indented particle. This can be seen in Fig. 2(a–c): although there is a small deviation from linearity at high values of $\frac{(1-{{\nu }_{\mathrm{m}}}^{2}){E}_{\mathrm{p}}}{\left(1-{{\nu }_{\mathrm{p}}}^{2}\right){E}_{\mathrm{m}}}$, this remains negligible and the observed behavior remains close to linear for all explored particle depths. Equation (19), which was derived by assimilating the particle to a punch pressed onto the matrix surface, thus leads to an appropriate functional description of indentation, even by deeply embedded non-conical indenters.

The value of the proportionality constant, however, appears to not only be different from unity (the expected value of $\alpha$ for a particle with axial symmetry acting as a rigid indenter) but also to vary with the depth to which the particle is embedded below the matrix surface. In practice this creates a difficulty, given that it is generally not possible to guess the particle depth or true diameter when viewing only its intersection with the free surface along which nanoindentations are conducted. Though not constant, however, the values of $\alpha$ vary within a relatively well-defined range, which for the studied cases is $0.5<\alpha <3$. We therefore propose that, if a large number of particles is tested within a sample and their depth into the matrix varies randomly, then by averaging data under the assumption that their depth relative to the plane of polish varies randomly, the proper materials properties can be obtained if an appropriate average value of $\alpha$ is used in data interpretation.

From the results of simulations performed here, calculated average values of $\alpha$ give $\overline{\alpha }=1.06$ for spherical particles with a coherent interface, $\overline{\alpha }=1.41$ for spherical particles with a frictionless interface, and $\overline{\alpha }=1.09$ for cylindrical particles with a coherent interface. The relative insensitivity of $\overline{\alpha }$ to the particle shape suggests that, if the particle can be taken to be well bonded to the matrix, then relatively accurate results might be obtained by taking $\overline{\alpha }$ only slightly above unity.

**Figure 3:**
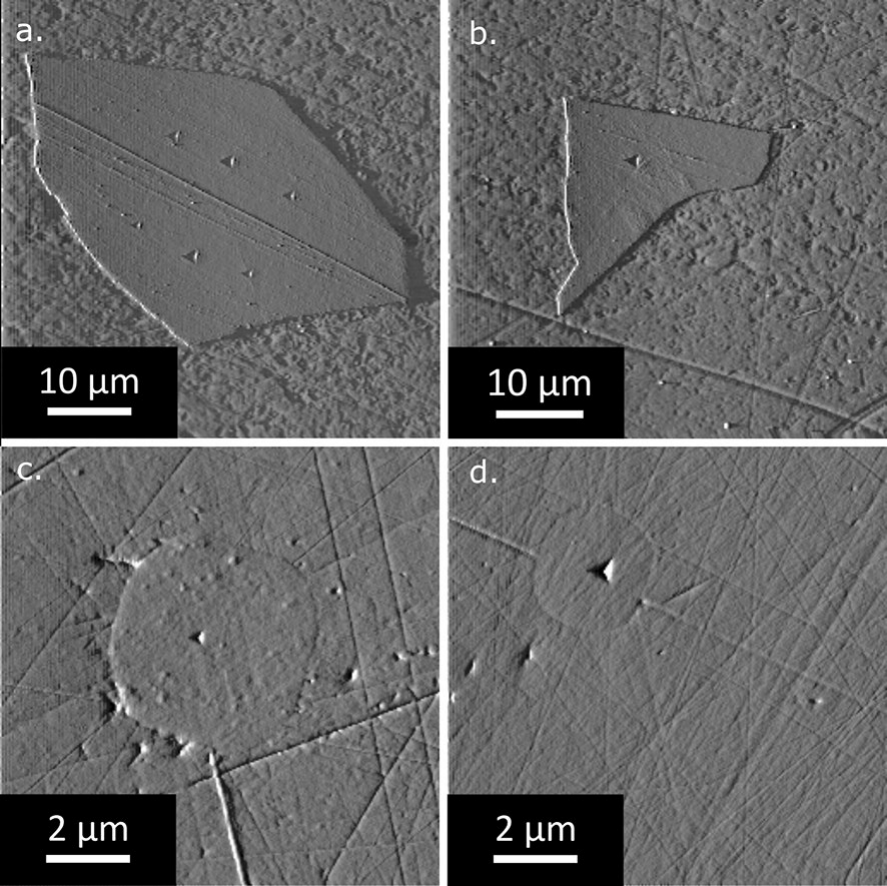
Images obtained by scanning the surface of probed particles with the Berkovich tip after indentation; (a) and (b) glass-slide fragments in epoxy resin, (c) and (d) silica inclusions in iron.

We test and illustrate this averaging approach using two composite systems. Both feature silica-based glass particles, which are embedded in a matrix of respectively higher and lower elastic modulus than the particles (Fig. 3). The volume fraction of particles in each system was not measured directly, but is sufficiently low that interactions between neighboring particles can be neglected and that the elastic modulus of material surrounding each particle can be taken to be that of the matrix.

For the first system, a composite material made of roughly equiaxed soda-lime glass fragments embedded in epoxy was fabricated by grinding an optical microscope glass slide with a mortar and pestle, and then sieving the powders to isolate particles retained between sieves with holes of side length 40 and 20 μm. The glass slide could be probed independently, such that the modulus of the particles was known and the method could be tested for its accuracy.

For the second system, roughly spherical silica inclusions 2 to 5 μm in diameter embedded in iron were tested (details of how the sample was fabricated can be found in the *Methods* section and in [19]). The nanoindentation results for those silica inclusions were compared to indentations performed on a fused silica reference sample used for calibration of the tip area function, assumed to come close to (but not necessarily to equal, given the density-dependence of silica glass properties) the silica making the inclusions.

**Figure 4:**
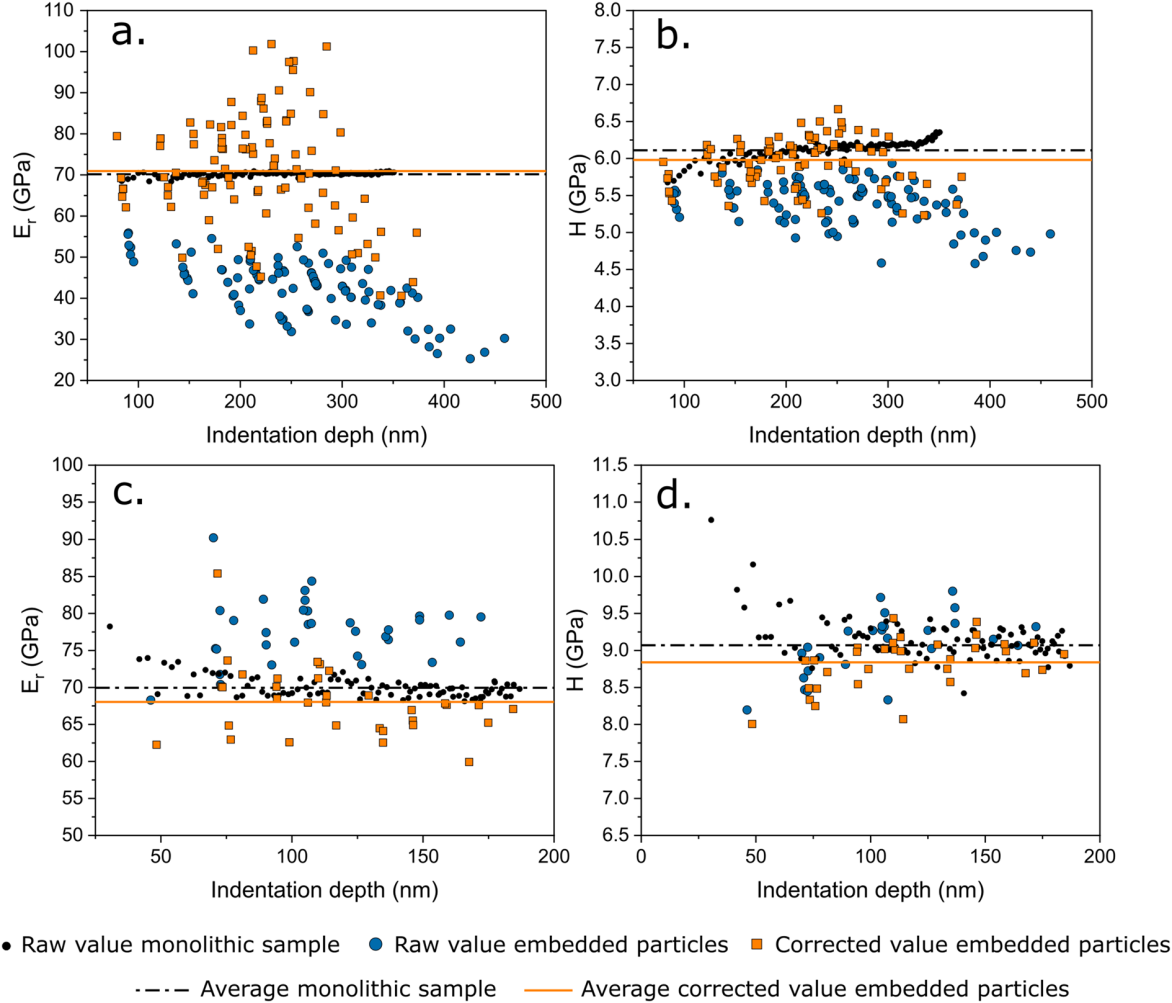
Reduced modulus and hardness values obtained by nanoindentations performed on (a) and (b) glass particles embedded in epoxy, and (c) and (d) silica inclusions in iron. Results after implementation of the present iterative correction procedure are compared with similar values obtained by indenting a monolithic sample with the same composition.

Measured hardness and reduced modulus values for indented particles, before (larger blue circles) and after (orange squares) the derived correction, are presented in Fig. 4, along with results obtained for their monolithic counterparts (smaller black circles). Orange data points for both systems in Fig. 4 are computed as if the particle modulus was unknown, meaning by iteration as described above (starting with the average apparent indented embedded particle modulus and iterating from there).

Two observations emerge. The first is that data are strongly dispersed, both before and after the correction. Corrected data can even on occasion [e.g., Fig. 4(a)] be more dispersed than are the raw data. A reason for this is that the correction of measured compliances tends to shift by a greater amount data points for which the measured stiffness is higher (see Eq. 14).

The second observation is that there is a good agreement between the *average* of corrected indented particle values and the results obtained from the monolithic samples. The dispersion in obtained modulus or hardness values measured by indenting embedded particles can be explained by (i) variations in particle morphology or depth, which affect, as shown above, the value of $\alpha$, and by (ii) variations in the particle size, which enters (for a given indentation) the extra compliance through the square root of the particle area along the surface of the sample (Eq. 13). By adopting a constant (average) value for alpha, the outcome is that corrected values are not less dispersed than raw values but display the right average after correction. Provided a large number of particles are tested and provided the depth at which those particles are intercepted by the plane of polish is random, then the average corrected value lies close to the real modulus (and hardness) of the particles, as can be observed in Fig. 4 by comparison of the average over all corrected data points (yellow continuous line, computed as if the particulate relative stiffness was unknown), and the average of values measured with the two particulate materials in bulk form (black dash-dotted line). We have not sought to evaluate the correlation between precision and number of particles tested; however, present data show that around 10% precision or better is obtained by testing 30 or more particles, whereas the average of raw data can be off by as much as 40% (see reduced modulus data in Fig. 4). It can also be noted in Fig. 4 that the elastic inhomogeneity has a larger influence on the apparent (uncorrected) elastic modulus than it has on the apparent hardness. This is not surprising since the matrix effect is primarily observed on the unloading stiffness. Still, since the stiffness enters the contact depth determination and hence the computation of the tip area function, if the extra compliance is large, then the induced variation in the contact area, and hence in the hardness, is also significant [Fig. 4(b)]. This holds even if the plastic deformation build-up around the indenter has not changed, and even if the matrix has remained in the elastic regime.

**Figure 5:**
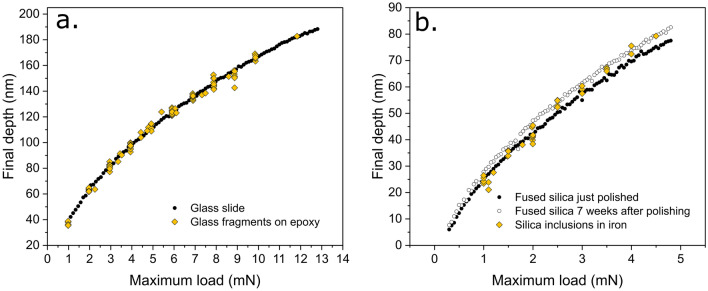
Final depth versus maximum load for indentations performed on: (a) soda-lime glass (glass slide and glass-slide fragments on epoxy) and (b) pure silica (fused silica and silica inclusions in iron).

Empirical evidence showing that the permanent deformation in the particle around the Berkovich indenter is not altered by the matrix can be found by comparing the final depths of indentations performed on embedded particles with those performed on their monolithic counterparts at equal peak load. This is because final depths, unlike (nanoindentation) hardness values, are not affected by the compliance of the system and give a true indication of permanent deformation processes under the indenter. Figure 5 presents final depth measurements on the embedded glass-slide fragments and on the silica inclusions compared with those obtained in their semi-infinite counterparts. For the glass fragments in epoxy, the final depths of indents performed on individual particles are in good agreement with those observed for its macroscopic counterpart. For silica inclusions, indentations on the fused silica reference sample were performed not only immediately after polishing the surface of the sample with a diamond suspension (down to 0.1 μm) but also, for the same sample, seven weeks after the polishing was performed. It is observed that final depths for indentations performed on silica inclusions generally lie between those observed for the just-polished sample and the “aged” surface, with the final depth in the latter always being slightly larger than in the freshly polished sample. This variation over time on the final depth observed in fused silica samples is associated with the formation of a soft hydrated layer on the surface of the silica, which leads to a small increase (2–4 nm) of the permanent deformation depth when the sample is left in an environment containing moisture [20, 21].

**Figure 6:**
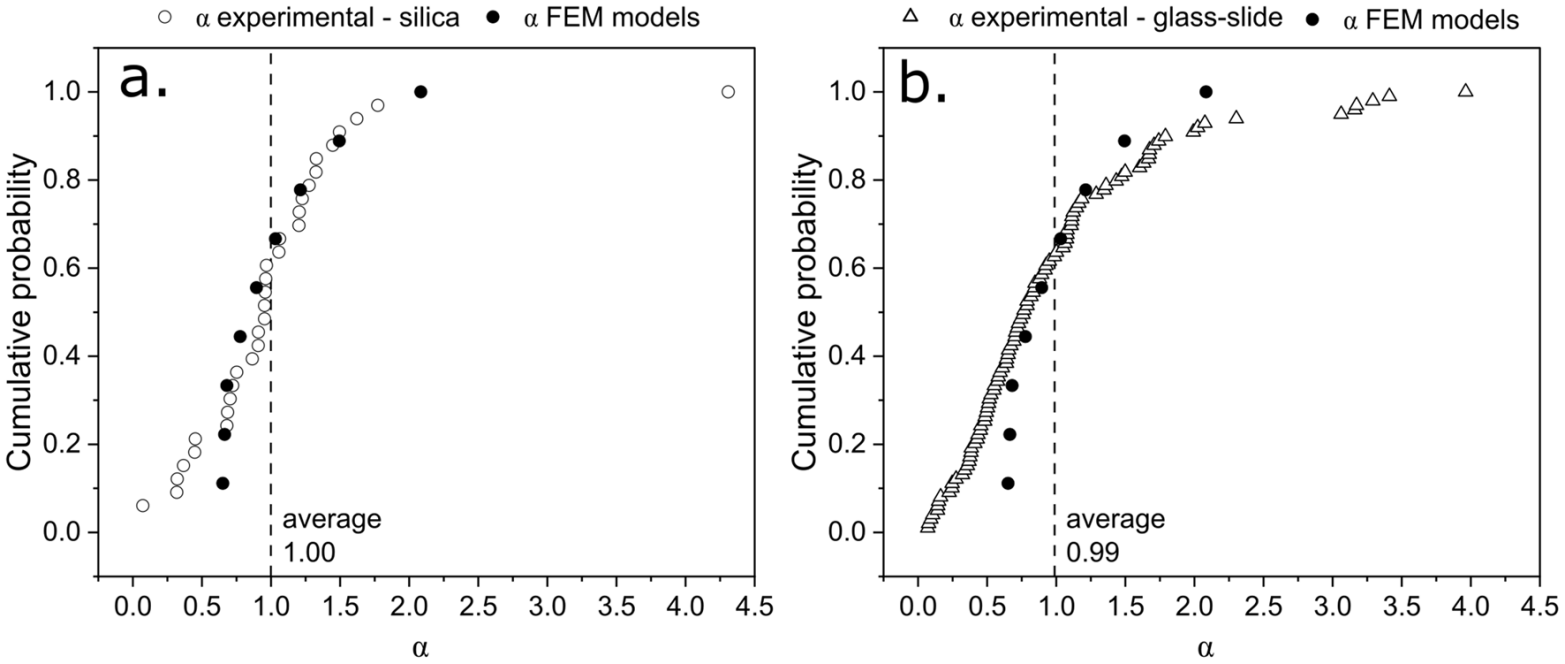
Cumulative probability function of alpha values obtained by finite element simulations (spherical particles fully bonded to the matrix shown here) compared with those obtained in (a) experiments performed on silica inclusions in iron and (b) glass-slide fragments in epoxy.

As a final check, the reduced modulus of the monolithic samples was used to back-calculate the value of alpha that would correspond to the deviation observed in each of the measurements performed on individual particles. Figure 6 shows the cumulative probability distribution function for (i) the values of (apparent, see Appendix A) alpha observed experimentally (open symbols) and for (ii) values calculated by finite element simulations (filled symbols). Exception made for tail ends of the distributions (which can be affected by a number of factors, such as an embedded particle depth so shallow as to affect particle indentation itself), the agreement is satisfactory, not only for the silica in iron inclusions [Fig. 6(a)], which are known to have (in most of the cases) a spherical morphology, but also for the glass fragments in epoxy, which have a morphology that clearly deviates from being spherical.

## Summary and conclusion

A method is proposed to correct data for the influence of elastic inhomogeneity when probing by nanoindentation particles embedded in a given different matrix, with a goal to enable the measurement of the reduced modulus and hardness of phases that are unavailable in bulk form, either because they are particulate or because they were precipitated within another, solid matrix phase. The method is defined by estimating the extra compliance (positive or negative) that is induced by the matrix in the nanoindentation signal.

Implementation of the method requires measurements of:

(i) The area of the particle along the surface on which indentations are placed;
(ii) The nanoindentation response of the particulate material to indentations separated from the particle/matrix interface by at least ten times the indentation depth and conducted on a sufficient number of particles (30 or more seems appropriate);
(iii) The elastic (or reduced) modulus of the matrix;
(iv) A first estimation of the elastic (or reduced) modulus of the indented particle. This can be obtained by averaging raw nanoindentation data collected on the embedded particulate material.

Provided those 30 or more particles are selected randomly and tested along a polished surface, then if Eq. (13) is applied with $\alpha =1.075$ on data from each particle and resulting values are averaged across all measurements, after iteration and convergence, the scheme will provide a reasonable correction for the influence of the matrix compliance on nanoindentation measurements, giving access to the intrinsic reduced modulus and hardness of the material making the particles. The method was implemented and its efficiency was demonstrated on two systems of opposite phase modulus contrast.

## Methods

### Finite element calculations

**Figure 7:**
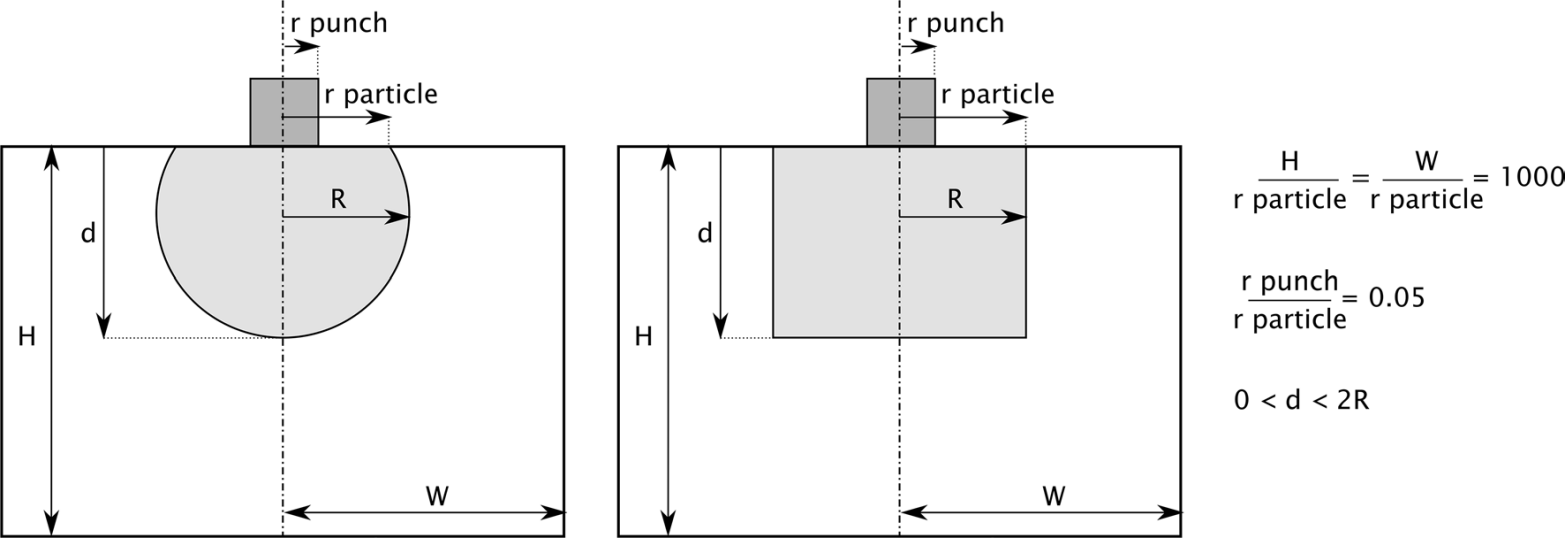
Schematic representation (not to scale) of the geometry and dimensions used for finite element simulations.

Finite element models were built using Abaqus/CAE 6.14-1 (Dassault Systèmes Simulia Corp., Providence, RI, USA, 2014). 2D axisymmetric models were built using CAX4R elements and the indenter probing the particle was modeled as an analytic rigid cylindrical punch. The particle and the surrounding continuum were modeled as isotropic linear elastic solids. Details of the geometry and dimensions of the models are presented in Fig. 7. The mesh was validated by comparing the results with the analytical solution for the compliance of a flat punch acting on a semi-infinite solid with homogeneous properties, refining the mesh until the deviation fell below 2%.

For the first round of models, perfect bonding was assumed between the particle and the matrix, the interface between the two being simply defined by an abrupt change in elastic properties. In order to explore the effect of the interface bond strength on the dimensionless compliance, a second round of models was run with all parameters remaining the same except for the properties of the interface between the particle and matrix, which was taken to be frictionless.

### Nanoindentation measurements

The silica in iron sample was fabricated by melting oxygen-containing high-purity iron and then deoxidizing the liquid metal with a Fe–5%Si prealloy. During deoxidation, the added silicon combines with the oxygen in the melt to form oxide inclusions, which remain in the metal structure after cool-down and solidification. More details of the sample fabrication can be found in Ref. [19].

All indentations were made using a Hysitron TI-950 apparatus (Bruker, Billerica, Massachusetts, US). Accurate positioning of the indents on the particles was possible thanks to the 500 nm XY axis resolution while positioning the indenter. The scanning capability of the instrument was used to provide an image of each tested particle respectively before and after the tests, of size 50 × 50 μm for the glass slide in epoxy and size 10 × 10 μm for the silica inclusions in iron. The area of the indented particles along the surface was measured from such images using the *Image J* software for image analysis [22]. For the monolithic samples (glass slide or silica calibration piece), arrays of > 100 indents were performed using a separation between indents greater than 50 μm.

The extra compliance, ∆*C,* was calculated using Eq. (13) with $\alpha =1.06$ for the silica inclusions in iron (based on the observation that those where generally spherical), and with $\alpha =1.075$ for the glass fragments embedded in epoxy, this being the average of calculated $\overline{\alpha }$ values for spherical and cylindrical particles strongly bonded to the matrix. Measured reduced modulus values were used instead of $\frac{E}{(1-{\nu }^{2})}$ in order to avoid assuming Poisson’s coefficients for the matrix or tested particles (note that the elastic properties of the indenter are also involved in computing the measured reduced modulus; however, its effect is canceled since it affects equally the particle and matrix material). An exception was made for the elastic properties of the metallic matrix, since pile-up development when indenting the metal leads to an underestimation of the contact area and an overestimation of the elastic modulus of the matrix. Elastic properties of the iron were therefore assumed to be $E=210$ GPa and $\nu =0.3$, which in combination with a diamond tip having $E=1140$ GPa and $\nu =0.07$ lead to a reduced modulus for iron of 192 GPa.

Equations (14) to (17) were used to compute the corrected modulus and corrected hardness. Once a corrected value for the reduced modulus was obtained, a new iteration for the calculation of the extra compliance (and other quantities) was made, since the corrected value represents a better estimation of the real modulus of the indented particles than the original, uncorrected, measurement. Iterations were repeated until the difference in reduced modulus between iterations (average values) was below 2 GPa. This was achieved in all cases after less than 3 iterations.

## Data Availability

The raw and processed data from this work are available for download from the Zenodo website at https://zenodo.org/ under the following digital object identifier (DOI): 10.5281/zenodo.6982946.

## Appendix A

In order to compute an experimental value of alpha for each indentation performed on a particle, the extra compliance associated with each single indentation was calculated using Eq. (10):

$$\Delta C={C}_{\mathrm{T}}-{C}_{\mathrm{A}},$$

with ${C}_{\mathrm{T}}$ the compliance of each indentation performed on a particle, and ${C}_{\mathrm{A}}$ the compliance of a similar indentation performed in its monolithic counterpart and at the same peak load. Each compliance value was simply computed as the inverse of the experimentally measured unloading contact stiffness.

Then simply reorganizing Eq. (13), the corresponding value of alpha was deduced as:

$$\alpha =\frac{\Delta C 2\sqrt{{A}_{\mathrm{c}}}}{\sqrt{\pi } \left(\frac{ \left(1-{{\nu }_{\mathrm{m}}}^{2}\right)}{{E}_{\mathrm{m}}}-\frac{ (1-{{\nu }_{\mathrm{p}}}^{2})}{{E}_{\mathrm{p}}}\right)},$$

with ${A}_{\mathrm{c}}$ the area of the particle measured along the surface of the sample.
